# Evaluation of breast calcif ication, calcif ication characteristics, and BI-RADS categories in patients with primary hyperparathyroidism

**DOI:** 10.20945/2359-4292-2026-0015

**Published:** 2026-02-16

**Authors:** Fatma Dilek Dellal Kahramanca, Sevgul Faki, Ekin Yigit Koroglu, Arzu Ozsoy, Ahmet Dirikoc, Oya Topaloglu, Reyhan Ersoy, Bekir Cakir

**Affiliations:** 1 University of Health Sciences, Ankara Bilkent City Hospital, Endocrinology Department, Ankara, Turkey; 2 Ankara Bilkent City Hospital, Endocrinology Department, Ankara, Turkey; 3 Ankara Bilkent City Hospital, Radiology Department, Ankara, Turkey; 4 Ankara Yildirim Beyazit University, Endocrinology Department, Ankara, Turkey

**Keywords:** Primary hyperparathyroidism, hypercalcemia, breast calcification, BI-RADS, breast cancer

## Abstract

**Objective:**

To determine the frequency and types of breast calcif ication, the distribution of breast imaging-reporting and data system (BI-RADS) scores, and the association between calcif ication and biochemical/clinical findings in patients with primary hyperparathyroidism (PHPT).

**Subjects and methods:**

We recruited ≥ 40-year-old female patients with PHPT (n = 104) and age-matched healthy women (n = 107) as controls. Mammography was performed on all participants. Calcif ication, calcif ication type, and BI-RADS scores were recorded, and patients were divided into two groups based on PHPT duration and presence/absence of calcification.

**Results:**

BI-RADS score distribution was indifferent between groups. The frequency of calcification and distribution of calcification types showed no difference between groups. Likewise, mammography findings were consistent among PHPT patients regardless of disease duration. There was no cutoff for disease duration that could predict the presence of calcification. Breast calcification was negatively correlated with parathyroid hormone (r = -0.220, p = 0.025) and 24-hour urine calcium levels (r = -0.195, p = 0.048), and positively correlated with age (r = 0.219, p = 0.025) in PHPT patients. Of the six patients who underwent cytological examination, one was found to be malignant (PHPT group).

**Conclusion:**

Female patients with PHPT do not have an increased incidence of breast calcification or higher BI-RADS scores compared to healthy women, and the calcification rates were unaffected by the duration of the disease. The presence of calcification does not appear to be associated with an increased risk of breast cancer in PHPT patients. Nonetheless, given the frequency of breast cancer and that the only patient with breast cancer was part of the PHPT group, it would be appropriate to screen these patients for breast cancer carefully.

## INTRODUCTION

Primary hyperparathyroidism (PHPT) is a disease characterized by the autonomous secretion of parathyroid hormone (PTH) from the parathyroid glands due to adenoma, hyperplasia, or carcinoma, despite normal or elevated serum calcium levels. The clinical manifestations of this disease primarily include nephrolithiasis-nephrocalcinosis, and osteoporosis. Additionally, disrupted calcium metabolism in PHPT ^(1)^ can lead to ectopic calcification in several organs, such as the skin ^(2)^, gallbladder ^(3)^, sclerochoroid ^(4-7)^, eyes ^(8)^, heart valves ^(9,10)^, myocardium ^(11)^, abdominal aorta ^(12)^, brain ^(13-15)^, cerebral gyrus ^(16)^, joint ^(11,17)^, lungs ^(18-20)^, pancreas ^(21)^, and stomach ^(22)^. The precise mechanism of this type of calcification is not fully understood; however, potential causes include increased levels of the calcium-phosphate product and the local secretion of free hydrogen ions, which result in an alkaline environment ^(18,22)^.

Breast calcif ication, commonly observed in mammography, can be present in both breast cancer and benign lesions. The morphology and distribution of calcifications are instrumental in evaluating whether breast lesions are benign or suspicious. Although studies ^(23-26)^, case series ^(27)^, and case reports ^(28-31)^ have documented breast calcification in patients with secondary hyperparathyroidism (SHPT), to our knowledge, there is no study examining its prevalence and characteristics in patients with PHPT. The incidence of breast cancer has been reported to be higher in patients with PHPT compared to those without PHPT. Hypothetically, this finding could be attributed to increased calcium deposition in the breasts and more frequent breast examinations in these patients ^(32)^. Given the above, we aimed to determine the frequency and characteristics of breast calcification, analyze the distribution of Breast Imaging-Reporting and Data System (BI-RADS) scores, and explore the relationship between calcification and biochemical or clinical features in patients with PHPT.

## SUBJECTS AND METHODS

Female patients aged ≥ 40 years old with PHPT and age- and body mass index (BMI)-matched healthy women were recruited. The healthy participants were selected from women referred to the radiology clinic for routine mammographic screening by physicians in family medicine, general surgery, and obstetrics and gynecology. These participants were matched for age, BMI, smoking status, menopausal status, and breast disease history. According to the American Cancer Society’s 2015 guideline update, women aged ≥ 40 years are recommended to undergo mammography ^(33)^.

PHPT was diagnosed based on either the presence of hypercalcemia along with elevated or inappropriately normal serum PTH levels, indicating overt PHPT, or normal serum calcium and 25-hydroxyvitamin (OH) D levels with elevated serum PTH and increased 24-hour urinary calcium/fractional excretion of calcium levels, indicating normocalcemic PHPT ^(9)^. Exclusion criteria included a PHPT duration of < 6 months, chronic kidney or liver disease, malignancy, pregnancy, lactation, history of breast surgery, history of chest radiotherapy, and use of medications affecting blood calcium levels (e.g., calcium supplements, thiazide diuretics, lithium, vitamin A, teriparatide, bisphosphonates, corticosteroids, rifampin, phenobarbital, phenytoin, calcitonin, chloroquine, plicamycin, etc.). Smoking habits, alcohol consumption, breast disease history, and family history of breast cancer were also recorded. Weight (kilograms) was divided by the square of height (meters) to determine the BMI.

Serum levels of calcium (normal: 8.5–10.4 mg/dL), albumin (normal: 3.2–4.8 g/dL), phosphate (normal: 2.4–5.1 mg/dL), magnesium (normal: 1.3–2.7 mg/dL), intact PTH (normal: 18.4–80.4 ng/L), 25 (OH) D (normal: 30–150 ng/mL), and alkaline phosphatase (normal: 42–98 U/L) were measured in the morning after an 8-hour fast and recorded. The 24-hour urinary calcium and fractional excretion of calcium were similarly documented. Corrected serum calcium was calculated using the formula: Corrected calcium (mg/dL) = [0.8 × (4 - serum albumin (g/dL))] + measured calcium (mg/dL). The highest recorded calcium and PTH levels were noted from the Ankara Bilkent City Hospital database or the national patient health database. Calcium × phosphate product levels and the highest such levels in the databases were also recorded. Hypercalcemia was classified as mild (10.5–11.9 mg/dL), moderate (12–13.9 mg/dL), or severe (≥ 14 mg/dL). PHPT duration was defined as the time between the first diagnosis and the date of mammography. The earliest available high serum calcium value in the hospital or national database was recorded as the time of initial diagnosis.

All participants underwent mammography using the Senographe Pristina Mammography system (GE Healthcare, Fairfield, Buc, France). Mammographic features were analyzed and reported according to the American College of Radiology’s BI-RADS by our radiologist (A.O.), who was blinded to the patients’ diagnoses. Calcification morphology was classified as benign or suspicious. Benign calcifications included skin, vascular, popcorn-like, large rod-like, round, rim, dystrophic, milk-of-calcium, and suture calcifications. Suspicious calcifications were described as amorphous, coarse heterogeneous, fine pleomorphic, or fine linear/linear branching. Additionally, the distribution of calcifications was classified as benign if diffuse, and as suspicious if linear or segmental ^(34)^.

Calcifications were recorded as unilateral or bilateral, indicating presence in one breast or both, respectively. Breast density was classified as A, B, C, or D, reflecting breast composition from almost entirely fatty to extremely dense, according to BI-RADS. Scores ranged from 0 to 6, where 0 required further imaging, 1 was negative, 2 was benign, 3 was probably benign, 4 was suspicious (with subcategories 4A, 4B, and 4C for low, intermediate, and high suspicion of malignancy, respectively), 5 was highly suggestive of malignancy, and 6 was a biopsy-proven malignancy ^(34)^. Participants were asked to return for further diagnostic evaluation when findings such as suspicious breast calcifications, asymmetric densities, or a mass were identified. If malignancy was suspected, a biopsy followed by surgery, if necessary, was suggested.

Dual energy X-ray absorptiometry was used to measure bone mineral density (BMD) in the femur, radius, and lumbar spine (L1–L4) with an anterior-posterior projection in both the patient and control groups (Lunar iDXA, GE Healthcare, USA). Postmenopausal subjects with a BMD T score of ≤ -2.5 were classified as having osteoporosis, those with a T score between -2.5 and -1 were classified as having osteopenia, and those with a T score ≥ -1 were categorized as normal. In premenopausal women, a BMD Z score of ≤ -2 indicated a bone density below the expected range for age, and > -2 was within the expected range. Premenopausal subjects with scores below the expected range were included in the osteoporosis group, whereas those within the expected range were considered to have normal BMD. Nephrolithiasis was defined as either a history of the condition or diagnosis by imaging modalities. If any, histopathological findings of parathyroidectomy with/without thyroidectomy as well as breast cytopathology were noted.

Patients and controls were compared with regard to demographic and laboratory data, as well as mammographic results, including calcification presence and types and BI-RADS scores, were compared between patients and controls. PHPT patients were further divided into groups based on disease duration of less than or more than 48 months and analyzed with respect to the same parameters. Additionally, patients with and without breast calcifications were compared.

This study was approved by the institution’s ethical committee (Date: January 18, 2021, no: 26379996/21, Chairman: Kara H.), in compliance with the Declaration of Helsinki. Informed consent was obtained from all patients and controls.

SPSS 24 package (IBM Corp., Armonk, NY, USA) was used for the statistical analysis. Descriptive statistics were given as mean ± standard deviation when normally distributed, and median (interquartile range 25%-75%) when not normally distributed. Nominal variables were given as percentage and number of cases. Chi-squared or Fisher exact test was used when categorical variables were compared. Student *t* test was used for parametric variables and Kruskal-Wallis test was used for nonparametric variables. Spearman’s or Pearson’s tests were used to assess correlation of breast calcification and laboratory or clinical variables. A *p* value < 0.05 was considered significant.

The sample size was determined for a comparison between two independent groups with PHPT and healthy controls. The calculation assumed a significance level (α) of 0.05, a statistical power (1–β) of 0.80, and an expected effect size (Cohen’s d) of 0.35, defined as the smallest clinically meaningful difference anticipated in the study. Under the assumptions of equal group sizes, approximate normal distribution, and homogeneous variances, the required sample size was 102 participants per group (204 in total). The Student’s *t*-test will be used for the primary comparison, while the Mann–Whitney U test will be applied if distributional assumptions are not met. All calculations were performed using G*Power version 3.1.

## RESULTS

Overall, 104 patients and 107 controls were included. Demographic and clinical findings, laboratory results, and imaging results are summarized in **Tables 1–3**. Age distribution and BMI were similar between both groups (*p* = 0.920 and *p* = 0.391, respectively). The median duration of PHPT was 48 months (min-max: 6–180 months). The frequency of current smoking was comparable between the patient and control groups. Regular alcohol consumption was observed in 3 patients and 1 control. The rates of previous benign breast disease and family breast cancer history were similar in both groups (*p* = 0.416 and *p* = 0.367, respectively). Menopausal status was also comparable (*p* = 0.066).

**Table 1.** Demographic and clinical data in patients with primary hyperparathyroidism and healthy control subjects; comparisons include primary hyperparathyroidism patients within and beyond 48 months, as well as primary hyperparathyroidism patients with and without breast calcification

Variables	PHPT (n = 104)	Control (n = 107)	*p*	PHPT duration within 48 months (n = 54)	PHPT duration more than 48 months (n = 50)	*p*	PHPT patients with calcification (n = 63)	PHPT patients without calcification (n = 41)	*p*
Age (years)	56 (50–62.8)	56 (50–63)	0.920	57.5 (50.75–63.25)	56 (50–62.3)	0.593	58.0 (53.0–63.0)	53 (49–60.5)	0.026
Height (cm)	157.8 ± 6.4	159.2 ± 6.6	0.122	157.0 ± 5.7	158.7 ± 7.1	0.171	157.32 ± 6.048	158.54 ± 6.92	0.363
Weight (kg)	75 (65–85)	74.5 (66.0–82.25)	0.945	75 (65–83.1)	72.2 (65.5–88.1)	0.699	72.1 (64.075–86.875)	76.0 (66.5–83.9)	0.754
BMI (kg/m^2)^	29.8 (25.7–34.1)	29.1 (26.2–32.5)	0.391	29.95 (26.88–33.48)	29.4 (25.3–34.7)	0.840	29.15 (25.575–34.875)	30.2 (26.75–32.65)	0.973
PHPT duration (months)	48 (24.3–69.5)						49.78 (26.00–74.00)	48 (17–67)	0.508
Current smoking (n = 206)	29 (27.9)	18 (17.6)	0.08	14 (25.9)	15 (30.0)	0.643	8 (12.7)	7 (17.1)	0.535
Breast disease history (n = 207)	15 (14.4)	11 (10.7)	0.416	10 (18.5)	5 (10.0)	0.217	10 (18.5)	5 (10.0)	0.217
Family breast cancer history (n = 206)	9 (8.7)	13 (12.6)	0.367	5 (9.3)	4 (8.2)	0.844	5 (9.3)	4 (8.2)	0.844
Menopausal status			0.066			0.491			0.113
Premenopause	20 (19.2)	11 (10.3)		9 (16.7)	11 (22.0)		9 (14.3)	11 (26.8)	
Peri- and postmenopause	84 (80.8)	96 (89.7)		45 (83.3)	39 (78.0)		54 (85.7)	30 (73.2)	

PHPT: primary hyperparathyroidism; BMI: body mass index.

**Table 2.** Laboratory results in patients with primary hyperparathyroidism compared to healthy control subjects. Comparison includes primary hyperparathyroidism patients assessed within 48 months and those assessed more than 48 months after diagnosis, as well as primary hyperparathyroidism patients with and without breast calcification

Variables	PHPT (n = 104)	Control (n = 107)	*p*	PHPT duration within 48 months (n = 54)	PHPT duration more than 48 months (n = 50)	*p*	PHPT patients with calcification (n = 63)	PHPT patients without calcification (n = 41)	*p*
Calcium (mg/dL)	11.1 (10.7–11.5)	9.6 (9.4–9.9)	<0.001	11.0 (10.7–11.3)	11.1 (10.8–11.9)	0.260	11.1 (10.7–11.5)	11.1 (10.75–11.4)	0.931
Highest calcium	11.6 (11.3–11.9)	9.9 (9.7–10.2)	<0.001	11.4 (11.1–11.7)	11.9 (11.5–12.3)	< 0.001	11.6 (11.29–11.90)	11.6 (11.24–12.01)	0.907
Calcium higher than 12 mg/dL	10 (9.6%)	0	0.001	0 (0.0%)	10 (20.0%)	0.001	5 (7.9%)	5 (12.2%)	0.472
Albumin (g/dL)	45 (43–47)	49.0 (45.0–45.4)	<0.001	45 (43.8–46.3)	45.0 (43.0–47.0)	0.832	45 (43.0–47.0)	40.04 (44.0–48.0)	0.267
Corrected calcium	10.65 (10.31–10.98)	9.21 (8.96–9.46)	<0.001	10.59 (10.30–10.93)	10.75 (10.32–11.14)	0.145	10.76 (10.32–11.02)	10.56 (10.30–10.97)	0.519
Highest corrected calcium	11.17 (10.87–11.52)	9.49 (9.23–9.79)	<0.001	10.98 (10.80–11.29)	11.42 (11.03–11.81)	<0.001	11.17 (10.88–11.50)	11.09 (10.77–11.65)	0.944
Phosphate (mg/dL)	2.8 ± 0.5	3.8 ± 0.5	<0.001	2.8 ± 0.5	2.7 ± 0.5	0.237	2.8 ± 0.5	2.7 ± 0.5	0.104
Calcium x phosphate (mg^2^/dL^2)^	29.43 (26.18–33.95)	35.35 (30.99–37.77)	<0.001	29.42 (26.24–33.97)	29.43 (25.43–33.56)	0.694	29.65 (26.71–33.98)	28.30 (24.70–32.90)	0.142
Highest calcium x phosphate (mg^2^/dL^2)^	30.69 (26.73–34.98)	35.96 (33.21–38.58)	<0.001	30.60 (26.92–34.86)	31.13 (26.56–36.01)	0.920	31.38 (28.65–36.00)	30.41 (25.71–34.69)	0.133
Magnesium (mg/dL)	2.1 (2.0–2.2)	2.0 (1.8–2.1)	<0.001	2.1 (2.0–2.2)	2.0 (2.0–2.2)	0.225	2.0 (1.9–2.2)	2.1 (2.0–2.3)	0.153
Alkaline phosphatase (U/L)	107 (87–131)	77 (67–93)	<0.001	105 (81.8–132)	107 (87.5–123.5)	0.868	107 (888.25–123.50)	107 (82.5–133.5)	0.745
Parathyroid hormone (ng/L)	156 (113.5–224.5)	52 (40–69)	<0.001	148.5 (111.8–208)	156 (120–261.3)	0.313	140.0 (112.0–204.0)	187 (131.5–258.5)	0.026
Highest parathyroid hormone	207.5 (155–285.1)	60 (43–75)	<0.001	193.5 (153.5–263)	225 (154.3–300)	0.416	187.0 (146.0–273.0)	235 (176–300)	0.104
25(OH) vitamin D (ng/mL)	39 (23–55.7)	48 (32–71.7)	0.001	37 (24.5–54.2)	39.5 (20.0–57.5)	735	40.0 (23.0–57.0)	37 (24.0–49.5)	0.630
Fractionated urinary calcium	0.021 (0.017–0.029)			0.0225 (0.0160–0.0290)	0.0200 (0.01775–0.02825)	0.545	0.0210 (0.0160–0.0280)	0.0220 (0.0180–0.02950)	0.196
Urinary calcium (mg/24h)	314.1 (230.0–428.1)			345 (203.7–491.3)	295.5 (233.8–411.5)	0.478	293 (202.72–411.0)	354 (271–470.5)	0.048
Urinary creatinine (mg/24h)	932.5 (737.0–1168.5)			985 (735.8–1177.5)	906 (731.8–1157.5)	0.569	917 (746.0–1151.0)	978 (704.5–1174.0)	0.793
Volume (mL)	2500.0 (1562.5–3475.0)			2600 (2000–3500)	2450 (1500–3425)	0.515	2600 (1500–3500)	2500 (1650–3325)	0.907

PHPT: primary hyperparathyroidism.

**Table 3.** Results of mammography and bone mineral densitometry in patients with primary hyperparathyroidism and healthy control subjects. Comparison includes primary hyperparathyroidism patients within 48 months and beyond 48 months since diagnosis, as well as primary hyperparathyroidism patients with and without breast calcification

Variables	PHPT (n = 104)	Control (n = 107)	*p*	PHPT duration within 48 months (n = 54)	PHPT duration more than 48 months (n = 50)	*p*	PHPT patients with calcification (n = 63)	PHPT patients without calcification (n = 41)	*p*
Breast density			0.625			0.256			0.422
A	8 (7.7)	14 (13.1)		3 (5.6)	5 (10.0)		4 (6.3)	4 (9.8)	
B	41 (39.4)	40 (37.4)		18 (33.3)	23 (46.0)		23 (36.5)	18 (43.9)	
C	44 (42.3)	41 (38.3)		25 (46.3)	19 (38.0)		27 (42.9)	17 (41.5)	
D	11 (10.6)	12 (11.2)		8 (14.8)	3 (6.0)		9 (14.3)	2 (4.9)	
BI-RADS			0.508			0.678			0.267
BI-RADS 0	78 (75.0)	74 (69.2)		39 (72.2)	39 (78.0)		48 (76.2)	30 (73.2)	
BI-RADS 1	5 (4.8)	8 (7.5)		2 (3.7)	3 (6.0)		1 (1.6)	4 (9.8)	
BI-RADS 2	17 (16.3)	23 (21.5)		11 (20.4)	6 (12.0)		11 (17.5)	6 (14.6)	
BI-RADS 3 and 4	4 (3.8)	2 (1.9)		2 (3.7)	2 (4.0)		3 (4.8)	1 (2.4)	
Presence of calcification	63 (60.6)	72 (67.3)	0.310	33 (61.1%)	30 (60.0)				
Localization of calcifications			0.392			0.599			
Unilateral	10 (15.9)	16 (21.6)		6 (18.2)	4 (13.3)				
Bilateral	53 (84.1)	58 (78.4)		27 (81.8)	26 (86.7)				
Nephrolithiasis	29 (27.9)	3 (2.8)	<0.001	9 (16.7)	20 (40.0)	0.908	20 (31.7)	9 (22.0)	0.276
Bone mineral density (n = 193)			<0.001			0.599			0.280
Normal	11 (10.7)	27 (30.0)	0.001	8 (14.8)	3 (6.1)		5 (7.9)	6 (15.0)	
Osteopenia	47 (45.6)	51 (56.7)	0.126	24 (44.4)	23 (46.9)		27 (42.9)	20 (50.0)	
Osteoporosis	45 (43.7)	12 (13.3)	<0.001	22 (40.7)	23 (46.9)	0.908	20 (31.7)	9 (22.0)	0.276

PHPT: Primary hyperparathyroidism, BI-RADS: Breast imaging-reporting and data system

Serum levels of calcium, corrected calcium, highest calcium, magnesium, alkaline phosphatase, and PTH were higher, while phosphate, albumin, calcium x phosphate, and highest calcium x phosphate levels were lower in the PHPT group than the control group (*p* < 0.001 for all). Similarly, the 25 (OH) vitamin D level was lower in the patient group (*p* = 0.001). The majority of PHPT patients (63.5%) had mild hypercalcemia, 9.6% had moderate hypercalcemia, and the remaining 26.9% had normocalcemic PHPT. The rates of nephrolithiasis and osteoporosis were higher in the PHPT group than in the control group (*p* < 0.001 for both).

**Figure 1:**
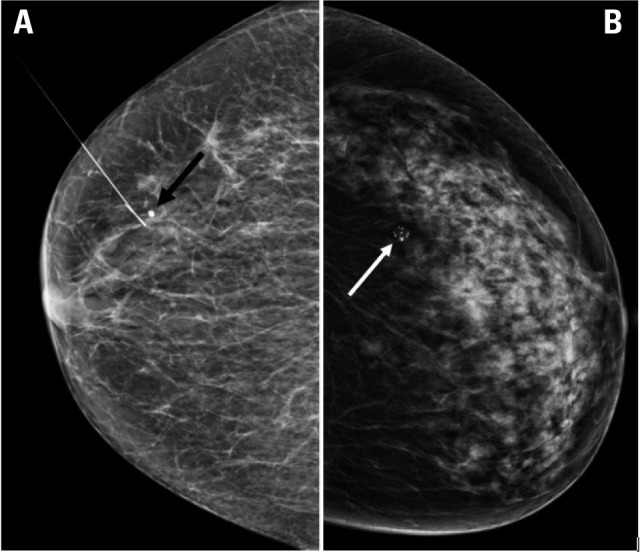
Mammographic findings in two patients with primary hyperparathyroidism showing suspicious breast calcifications. (**A**) Punctate calcifications are clustered in the upper outer quadrant of the right breast, identified as suspicious (at the tip of the localization wire’s hook). The punctate calcification indicated by the black arrow is benign. (**B**) Pleomorphic microcalcifications formed in clusters in the central section of the outer quadrant of the left breast are indicated as suspicious (white arrow).

The BI-RADS score did not differ, with BI-RADS 0 being the most prevalent score in both groups. Calcifications were observed in 63 (60.6%) patients with PHPT and 72 (67.3%) individuals in the control group. In the PHPT group, 1 patient had suspicious calcifications, 1 patient had both benign and suspicious calcifications (**Figure 1**), and the rest had benign calcifications on mammography. All breast calcifications were benign in the control group. Calcifications were mostly bilateral in both groups (*p* = 0.392).

Parathyroidectomy was performed on 63 patients (60.6%). Except for one patient with atypical parathyroid adenoma, all patients had histopathologically confirmed parathyroid adenoma. Concomitant thyroidectomy was performed on 19 (18.3%) patients, revealing papillary thyroid carcinoma in 12 patients, noninvasive follicular thyroid neoplasm with papillary-like nuclear features (NIFT-P) in 1 patient, concurrent papillary thyroid carcinoma and NIFT-P in 2 patients, and benign findings in 4 patients.

PHPT patients were categorized based on the presence (n = 63, 60.6%) or absence (n = 41, 39.4%) of breast calcification. PTH and urinary calcium levels were lower, and age was higher in PHPT patients with breast calcification than those without calcification (*p* = 0.026, *p* = 0.048, and *p* = 0.026, respectively). Other clinical and laboratory variables were similar in both groups. A weak correlation was found between breast calcification and age (r = 0.219, *p* = 0.025), PTH (r = -0.220, *p* = 0.025), and 24-hour urinary calcium levels (r = -0.195, *p* = 0.048) in PHPT patients. There was no cut-off for disease duration that could predict the presence of calcification. The area under the ROC curve was 0.534 (95% CI: 0.456–0.612, *p* = 0.400), indicating no significance.

PHPT patients were divided based on a disease duration of less than or greater than 48 months, the median duration. Both groups were similar regarding the frequency and localization of calcification, BI-RADS score, breast density, history of benign breast disease, and family breast cancer history (*p* > 0.05 for all). Similarly, age, BMI, current smoking status, menopausal status, and BMD,=, along with laboratory parameters except for the highest calcium level, were comparable between the two groups (*p* > 0.05 for all). The highest calcium level and nephrolithiasis frequency were more prominent in the longer duration group than the shorter duration one (*p* < 0.001 and *p* = 0.008, respectively). All patients with calcium levels of > 12 mg/dL were in the longer duration group. One patient with suspicious calcification was in the shorter duration group, and one with both suspicious and benign calcification was in the longer duration group; others had benign calcifications.

Biopsy referral rates were 1.9% (2/104) in PHPT patients and 3.7% (4/107) in the control group. Two PHPT patients with suspicious calcifications underwent excisional breast biopsy, resulting in one invasive breast cancer and one atypical ductal hyperplasia diagnosis. Three controls underwent tru-cut biopsy, and one underwent excisional biopsy, with all 4 controls having benign cytology.

## DISCUSSION

The rate and type of breast calcification, as well as the distribution of BI-RADS scores, were similar in female patients with and without PHPT. Patients with disease durations both shorter and longer than 48 months also exhibited similar mammographic findings. These results may suggest that the presence of calcification is not associated with an increased incidence of breast cancer in PHPT patients. In the patient group, breast calcification was positively correlated with age and negatively correlated with PTH and 24-hour urine calcium levels.

Several studies have explored the relationship between PHPT and breast cancer. Both conditions exhibit similar sex and age associations ^(35)^ and have shown a steadily increased incidence over the past 40 years ^(32)^. Breast cancer is the most frequent cancer in women and is more common in women than in men, similarly to PHPT ^(36,37)^. The frequency of the association between breast cancer and PHPT was found to be similar to that of the general population in one study ^(38)^. Conversely, in a large-scale cohort study comparing the incidence of subsequent malignancy in patients who underwent surgery for PHPT with those who did not, PHPT was associated with an increased risk of developing cancer (e.g., kidney, colon, and squamous cell skin cancer), particularly breast cancer. This risk remains consistent over time and does not diminish even 15 years post-operation. Therefore, the increased risk of malignancy is not due to the biochemical changes in PHPT, but likely results from shared genetic and environmental etiological factors ^(39)^. These common factors are considered to be ionizing radiation and hypercalcemia. Another reported risk factor associated with a higher incidence of both diseases is being overweight ^(40)^.

Although there is no literature examining hypercalcemia-specific breast tissue, it is hypothesized that calcium accumulation in the breast may be more pronounced in PHPT, leading to an increased diagnosis of breast cancer due to more frequent breast-focused examinations ^(32)^. To determine whether calcium accumulation in breast tissue increases in PHPT, we designed this study. We observed similar frequencies and morphologies of breast calcification, as well as BI-RADS scores in PHPT patients and controls. Therefore, we conclude that the higher prevalence of breast cancer among PHPT patients, as suggested in the literature, may not be attributed to calcification presence. On the other hand, we identified one malignant and one atypical ductal hyperplasia case, both in the PHPT group, while all four cases in the control group were benign. Although there is no statistically significant difference between patient and control groups, we believe that PHPT patients should be carefully examined for breast cancer as both malignant and atypical cytological findings were present in this group.

Examining the inverse relationship, the frequency of PHPT in breast cancer patients was higher than its incidence in the healthy female population ^(35,41)^. In non-aggressive breast cancer patients without evidence of PHPT, significantly higher calcium and PTH levels were noted, regardless of clinical stage and antitumor treatment, compared to the healthy controls ^(41)^. The prognosis of breast cancer patients, irrespective of previous PHPT operations, was found to be similar ^(40)^.

Although no study exists in the literature examining breast tissue calcification in PHPT patients, there are three studies concerning patients with SHPT due to chronic kidney disease or end-stage renal disease ^(23-25)^. An increase of up to 94% in breast calcification rates has been noted in patients with SHPT associated with chronic renal failure, specifically among those on long-term hemodialysis ^(25)^. The increased calcification in SHPT compared to the general population is thought to relate to decreased phosphate excretion and rising levels of serum calcium-phosphate product, though it likely results from multiple factors, including the duration of renal failure, dialysate calcium concentration, tissue pH, calcium, magnesium and fluoride levels, metabolic acidosis, hyperoxalemia, hyperlipidemia, and corticosteroid treatment. Calcifications occurred in both breasts in 98% of SHPT patients ^(23)^.

Correlation between calcification and either serum PTH levels or the calcium-phosphate product SHPT has been inconsistent across studies. Breast calcifications associated with SHPT are benign, not linked with breast masses, and should not be confused with suspicious calcifications indicative of breast cancer, according to earlier studies ^(23,25)^. These studies, however, did not include data on BI-RADS, callback rates for additional diagnostic workups, biopsy recommendation rates, clinical outcomes related to increasing calcifications, or the incidence of suspicious calcifications. A more recent study by Castellanos and cols. ^(24)^ found that while increased breast calcification is generally benign, the incidence of malignancy-associated calcifications is slightly higher in SHPT patients compared to a control group. Consequently, these patients have a higher likelihood of being referred for a biopsy if recalled for further diagnostic evaluation ^(24)^. None of the three studies reported breast cytology results, leaving a gap in the literature on whether women with SHPT have a higher incidence of breast cancer.

Contrary to these findings in SHPT patients, our study did not detect an increased rate of breast calcification in PHPT patients. Similar to findings in SHPT studies, most calcifications in PHPT were benign and bilateral. Only two participants, both with PHPT, had suspicious calcifications. One potential explanation for the differing calcification rates between PHPT and SHPT could be the comparatively lower phosphate and calcium-phosphate product levels in PHPT patients, as opposed to SHPT patients. Despite a reduction in phosphate levels, the calcium-phosphate product may still be high in PHPT due to elevated calcium levels from increased bone resorption, particularly in severe hypercalcemia. The role of the calcium-phosphate product in PHPT remains unclear, despite its widespread application in chronic renal disease management. Few studies have explored its level in PHPT patients, with some linking it to renal dysfunction but not to aortic valve calcification ^(9,42)^. Lower product level may be related to the reduced phosphate levels and the modestly elevated or even normal calcium levels observed in most of our patients. Another possibility regarding the differences in PHPT and SHPT calcification rates is that the microenvironment of breast tissue in PHPT patients differs from systemic levels in a manner that promotes calcification.

In our study, we found a negative correlation between calcification in PHPT patients and PTH levels, in contrast to the study of Sommer and cols. ^(23)^ involving patients with renal hyperparathyroidism, which showed a positive correlation. We additionally observed positive correlations of breast calcification with age and a negative correlation with urinary calcium levels. The frequency of breast calcification is known to increase with age, and microcalcification is more common in older patients ^(43)^. However, our evaluation of PHPT patients concerning breast calcification and its related factors is the first in the literature. Further research is needed to elucidate whether renal dysfunction, more severe hypercalcemia/hypophosphatemia, higher PTH levels, or other factors might affect calcification rates in PHPT patients.

Several studies have compared laboratory and demographic characteristics in PHPT patients with and without calcifications in various tissues or organs. The severity of abdominal aortic calcification in PHPT and breast calcification frequencies in SHPT correlate with PTH levels ^(12,23)^. Patients with mild PHPT have shown increased calcification area in the aortic valve, positively and independently associated with PTH levels ^(9)^. Pepe and cols. ^(12)^ reported that older age, years since menopause, and time since diagnosis as distinguishing factors between PHPT patients with and without abdominal aortic calcification. In another study, age was the sole predictor of gallstone development in PHPT patients ^(3)^. Normocalcemic PHPT patients with renal calcifications had increased serum PTH, 1.25(OH)_2_ vitamin D, and urinary calcium compared to those without calcifications ^(44)^. Contrary to this study, Perez and cols. ^(45)^ reported that PHPT patients with nephrolithiasis had similar serum calcium and PTH levels compared to patients without nephrolithiasis, although urinary calcium levels were increased. Ejlsmark-Svensson ^(46)^ found that renal calcification correlated with the severity of PHPT as measured by PTH levels, 24-hour urine calcium, and the degree of hypercalcemia ^(46)^.

It is suggested that increased serum calcium levels due to the oversecretion of PTH may lead to calcium deposits in breast ducts ^(47)^. In our study, we did not find an increased rate of calcification in PHPT patients compared to the control group. Nevertheless, PHPT patients with breast calcification had lower PTH and urinary calcium levels but were older compared to those without calcification. Additionally, as noted earlier, calcification in PHPT patients correlated positively with age and negatively with PTH levels and 24-hour urinary calcium levels. Differences in the physiological characteristics of various tissues, the variable effects of PTH on these organs or tissues, the selection of different patient groups (e.g., normocalcemic PHPT, mild PHPT, asymptomatic PHPT), and diagnostic criteria (e.g., imaging methods, serum calcium range) may explain the diversity of results in the literature ^(35)^.

The duration of PHPT can affect the course of some clinical and laboratory parameters. PHPT patients managed nonoperatively showed an increased annual rate of renal stone events in the year before and after diagnosis, with the rate decreasing after five years ^(48)^. Some studies have shown that a proportion of patients with normocalcemic PHPT progressed to hypercalcemia over time, while other studies did not find this progression ^(49)^. In our study, nephrolithiasis was more frequent, and the highest calcium levels were more prominent in the group with longer disease duration compared to the group with shorter duration.

Despite our promising findings, this study has some limitations. Due to the increasing access to laboratory and imaging modalities, patients are diagnosed at earlier stages in recent years. Consequently, most of the patients in the study had mild hypercalcemia. This may have contributed to possibly lower rates of calcium-phosphate product levels and, therefore, a lower rate of breast calcification. Additionally, the cross-sectional design might have resulted in a low number of breast cancer patients. Given the often asymptomatic or subclinical nature of PHPT, underestimation of the disease duration is possible in the current study. Consequently, the disease duration may be inaccurately short in some cases, possibly because the disease started earlier than the first record available in the hospital database. This may affect some analyses performed based on disease duration, particularly its effect on the frequency of breast calcification. The lack of data on ionized calcium measurements, calcification subtypes, and the physical examination findings of the breast were other limitations of the study.

In conclusion, it can be inferred from this study that breast calcification may not be considered a target organ involvement, and the presence of calcification is not related to an increased incidence of breast cancer in PHPT patients. Although not statistically significant, the fact that all patients with non-benign cytology results were in the PHPT group may suggest that PHPT patients should be carefully screened for breast cancer. Nonetheless, these results should be confirmed in patients with different levels of hypercalcemia, in different age groups, and among patients with longer follow-up periods and a larger cohort.

## Data Availability

datasets related to this article will be available upon request to the corresponding author.
